# Therapeutic potential of endometrial stem cells encapsulated in alginate/gelatin hydrogel to treat of polycystic ovary syndrome

**DOI:** 10.1016/j.reth.2024.08.016

**Published:** 2024-08-30

**Authors:** Fatemeh Kouchakzadeh, Somayeh Ebrahimi-Barough, Behrouz Aflatoonian, Jafar Ai, Fahime Mazaheri, Fateme Montazeri, Fatemeh Hajizadeh-Tafti, Jalal Golzadeh, Reza Naser, Masoumeh Sepehri, Seyed Mehdi Kalantar

**Affiliations:** a Department of Tissue Engineering, School of Advanced Technologies in Medicine, Tehran University of Medical Sciences, Tehran, Iran; b Stem Cell Biology Research Center, Yazd Reproductive Sciences Institute, Shahid Sadoughi University of Medical Sciences, Yazd, Iran; c Medical Nanotechnology and Tissue Engineering Research Centre, Yazd Reproductive Science Institute, Shahid Sadoughi University of Medical Sciences, Yazd, Iran; d Abortion Research Center, Yazd Reproductive Sciences Institute, Shahid Sadoughi University of Medical Sciences and Health Services, Yazd, Iran

**Keywords:** Polycystic ovary syndrome, Endometrial stem cells, Injectable alginate/gelatin hydrogel, Clomiphene citrate, Intraovarian injection, Fertility

## Abstract

Polycystic ovary syndrome (PCOS) is a prevalent endocrine disorder in women, often leading to infertility due to anovulation. Recent advances suggest that endometrial stem cells (EnSCs) hold considerable promise for tissue regeneration, which could be pivotal in treating PCOS. To enhance the survival and stabilization of EnSCs within the ovary, the EnSCs were encapsulated in an injectable alginate/gelatin hydrogel (SC–H), which has excellent biocompatibility to support the survival of EnSCs. Polycystic ovary syndrome was induced in female Wistar rats using intraperitoneal injection of letrozole over 21 days. Then the rats were treated with SC, SC-H and clomiphene citrate for one-month post-PCOS induction. The effects of these treatments were evaluated based on changes in body and ovarian weights, inflammatory markers, endocrine profiles, and ovarian histology. The Induction of PCOS led to a significant increase in body and ovarian cyst weight, elevated serum levels of testosterone, luteinizing hormone (LH), and anti-Müllerian hormone (AMH), alongside reduced follicle-stimulating hormone (FSH) and progesterone levels. Histologically, there was a decrease in granulosa cells, immature follicles, and corpus luteum numbers. Treatment with SC and SC-H significantly mitigated these alterations, indicating improved PCOS conditions. Our findings demonstrate that SC and SC-H treatments can effectively ameliorate the symptoms of letrozole-induced PCOS in rats, primarily through their anti-inflammatory effects. This study lays the groundwork for potential clinical applications of EnSCs encapsulated in alginate/gelatin hydrogel as a novel therapeutic strategy for PCOS, highlighting the importance of biomaterials in stem cell-based therapies.

## 1 Introduction

Polycystic ovary syndrome (PCOS) is the most common reproductive and endocrine syndrome, affecting 10–18% of women of reproductive age worldwide [1]. It is characterized by hyperandrogenism, ovulatory dysfunction, and polycystic ovaries, leading to potential long-term health issues like type 2 diabetes, cardiovascular diseases, and endometrial cancer [2]. Women with PCOS have an increased Gonadotropin hormone-releasing hormone (GnRH) pulse frequency, which causes higher LH and androgen production and a relative decrease in FSH, disrupting egg maturation and leading to the formation of ovarian cysts. These cysts contribute to hormonal imbalances and ovulation issues [[3], [4], [5]]. Persistent low-level systemic inflammation is also a feature of PCOS, with elevated levels of inflammatory proteins such as Interleukin-6 (IL-6), Tumor necrosis factor alpha (TNF-α), Interleukin-1 (IL-1), and C-reactive protein (CRP), which can impair ovarian function and fertility [5]. Elevated androgen levels in PCOS can suppress certain T-helper cells and promote regulatory T cells, exacerbating inflammation [2]. Interestingly, reducing androgens does not seem to alleviate inflammation, but anti-inflammatory treatments may reduce androgen levels and improve ovulation, especially in insulin-sensitive women with PCOS [5]. Current treatments for PCOS, which aim to regulate menstrual cycles, reduce testosterone levels, and stimulate ovulation, often come with adverse side effects, highlighting the need for new therapeutic approaches. Regenerative medicine offers a promising alternative, particularly through the use of mesenchymal stem cells (MSCs) and endometrial stem cells (EnSCs). MSCs have been shown to possess anti-fibrogenic, antioxidant, anti-inflammatory, and regenerative properties, as well as immunomodulatory effects that may correct immune-related abnormalities in reproductive disorders [[6], [7], [8]]. MSC transplantation in PCOS patients has improved ovarian function, reduced inflammation, and balanced hormone levels [[9], [10], [11]]. EnSCs, derived from the uterine lining, are easily accessible and pose a low risk of rejection, making them a valuable resource in reproductive medicine [8,12].

The human endometrium is a dynamic tissue that undergoes cycles of growth and regression with each menstrual cycle. Endometrial regeneration also occurs following childbirth and extensive excision and occurs in postmenopausal women receiving estrogen replacement therapy. It is likely that mature stem/progenitor cells are responsible for this remarkable regenerative capacity. Stem cells derived from endometrium have a unique potential as therapeutic agents such as autologous transplantation due to non-invasive access, ease of isolation, lack of ethical problems and high clonability [13]. Therefore, endometrium may be an alternative source of MSC-like cells for tissue engineering purposes without additional complications compared to other stem cell sources. Transplantation of endometrial-derived stem cells is logically thought to show superior therapeutic effects for female patients with reproductive dysfunction due to the geographical relationship in which the cells originate from the endometrium. Therefore, the persistence of stem cells in the reproductive organs (uterus and ovary) of homologous receptors is likely to be higher, which provides an excellent opportunity to heal the damaged ovary. It also showed that granulosa cells are able to promote the differentiation of endometrial-derived stem cells into oocyte-like cells in vitro. In addition, subsequent studies also confirmed that endometrial-derived stem cells showed significant improvement in ovarian function, possibly due to improved germ stem cell renewal, promoting ovarian cell proliferation and follicle growth, while inhibiting follicle apoptosis and ovarian fibrosis [14,15].

Currently, two types of stem cells obtained from the endometrium, including menstrual blood-derived stem cells (MenSCs) and endometrial stem cells (EnSCs), have received much attention. In the only study that has been conducted in the field of PCOS treatment with cells derived from the endometrium, stem cells derived from human menstrual blood were used. Based on the results of this study, stem cells derived from human menstrual blood can improve PCOS symptoms by improving folliculogenesis, reducing ovarian and body weight, collagen distribution and vascular volume. There is still no study on the use of endometrial stem cells for the treatment of PCOS, but it is expected to work like MenSCs due to the similarity of origin and function. EnSCs were used instead of MenSCs in this study due to the possibility of fungal and bacterial contamination of menstrual blood samples during sampling [16,17].

In the realm of regenerative medicine, cell transplantation via injection is an appealing, minimally invasive treatment option that does not require surgery [18,19]. To overcome the challenge of low survival rates of directly injected cells, hydrogels have been developed as carriers to enhance cell retention and survival at the injection site [20]. Injectable hydrogels, particularly those made from alginate/gelatin, are advantageous due to their biocompatibility, mechanical properties, and ability to form a gel in situ, providing a supportive matrix for cell culture and transplantation. This combination of regenerative medicine techniques and hydrogel technology represents a cutting-edge direction for PCOS treatment strategies [21].

In a recent study, the impact of endometrial stem cells, both alone and encapsulated in an injectable alginate/gelatin hydrogel, was investigated in female rats with PCOS. The study focused on assessing changes in weight, tissue characteristics, hormonal fluctuations, and serum levels of inflammatory factors. This research could pave the way for new treatments that leverage the regenerative potential of stem cells and the supportive properties of hydrogels to address the complex challenges of PCOS.

## 2 Materials and methods

### 2.1 Ethics

All animal experiments were approved by the Ethics Committee of Tehran University of Medical Sciences (Ethical Approval No. IR.TUMS.AEC.1401.104) in accordance with institutional and international guidelines. 8-week-old female rats were purchased from Yazd Reproductive Sciences Institute. The conditions of the rats were maintained in free food and water conditions, 12 h of light/12 h of darkness during 24 h and at room temperature [22].

### 2.2 Endometrial stem cells isolation and characterization

#### 2.2.1 Endometrial stem cells isolation

Human endometrial stem cells were isolated and characterized as explained elsewhere [17]. In sum, in this study, healthy women aged 20–35 years in the secretory phase of their menstrual cycle provided endometrial biopsies. These samples were transported in Hank's medium supplemented with 1% Penicillin/Streptomycin (Pen/Strep) and 1 μg/ml amphotericin B. Upon arrival at the laboratory, the endometrial tissue was placed in a sterile 10 cm Petri dish containing warm Hank's medium with the same supplements, serving as a wash solution. The tissue was then finely minced into 1–2 mm fragments using a sharp scalpel. Subsequently, the minced tissue was transferred into sterile 15 ml Falcon tubes filled with 1 mg/ml collagenase I and incubated at 37 °C for 30–45 min. After incubation time, pre-warmed sterile complete medium (DMEM/F12 + FBS10%, 1% Pen/Strep) was added to neutralize the collagenase I. To separate the glandular epithelial components, it was passed through a 70 μm Falcon cell strainer once and twice through a 40 μm Falcon cell strainer. The cells that passed through the filter were centrifuged at 300×*g* for 10 min. The supernatant was discarded, and the cell pellet was resuspended in 1 mL of pre-warmed plating medium (DMEM-F12 with 15% FBS, 1% Pen/Strep, 1% glutamine, and 1 μg/mL amphotericin B). This cell suspension was added to culture flasks along with an additional 2.5 ml of plating medium and incubated for 24 h. The following day, an extra 3 ml of plating medium was added, and the cells were cultured for a week in a cell incubator. Once the cells reached 90% confluence, they were harvested for the first passage. Cells from the third passage were then utilized for the animal study [23].

#### 2.2.2 Immunophenotyping analysis

The phenotype characterization of the passage three (P3) EnSCs was performed by flow cytometry (FACCS Calibure, BD bioscience San Jose, CA, USA) for surface markers with the fluorescein isothiocyanate- or phycoerythrin - or peridinin chlorophyll protein complex conjugated anti-(CD90, CD146, CD105, CD31 and CD34) monoclonal antibodies. After adding the antibody, the obtained cell suspension (1 × 10^6^ cells) was washed twice with PBS. Then, the cell suspension was incubated for 30 min at 4 °C with monoclonal antibodies in the dark. Finally, the samples were washed with PBS and analyzed in Cytomics FC 500 MPL cytometer (Beckman Coulter, USA) [22,24].

#### 2.2.3 In vitro differentiation of human EnSCs into adipocytes and Osteocytes

Adipogenic and osteogenic differentiation was performed and analyzed as reported. Briefly, for the differentiation assay, P3 EnSCs suspended in growth medium were seeded at a density of 2 × 10^4^ cells/well in a 6-well plate and grown to confluence. After that, the growth medium was replaced with adipose differentiation medium or osteogenic differentiation medium, and the induction medium was replaced every 3 days. The preparation steps of control cells were similar to P3 EnSCs except that they were cultured only in the growth medium. At the end of the induction period, the cells were washed and fixed. Osteogenic differentiation was confirmed by alizarin red staining and Adipogenic differentiation was confirmed by Oil red staining [25].

#### 2.2.4 G-banded karyotyping protocol

Karyotypes were analyzed in cultured endometrial stem cells using chromosome G-banding method. To 300 μL of cell suspension with a density of 2 × 10^4^/ml, 100 μL of colchicine with a concentration of 40 μg/ml was added. The cells were incubated for 4 h in an incubator and transferred to a 15 ml centrifuge tube. After centrifugation for 8 min at 182×*g*, the culture medium containing colchicine was separated and 4 ml of 0.075% KC1 was added instead. The sample was incubated for 5 min and then 2 ml of Carnoy's solution (absolute ethanol: 1:1 ethanol: glacial acetic acid) was added. The sample was incubated for 5 min and centrifuged again for another 8 min at 182×*g*. The culture medium was removed and 4 ml of Carnoy's fixative was added to it. Cells were incubated and centrifuged again, which was repeated twice. DMEM/F12 medium containing 10% FBS was added to the cell sample and a small amount of the suspension was poured on the slides. After two days at room temperature, the slides were placed in a slide dryer at 75 °C for 4 h. Finally, Giemsa staining was done for 15 min and karyotypes of generations were analyzed using G-banding technique [26].

### 2.3 Scaffold preparation and characterization

#### 2.3.1 Preparation of injectable alginate/gelatin hydrogel

Sodium alginate (low viscosity derived from brown algae), Gelatin (type B from bovine skin), sodium chloride (NaCl), transglutaminase enzyme were acquired from Merck and Sigma-Aldrich company. The preparation of the injectable alginate/gelatin hydrogel was carried out in accordance with methodologies described in previous studies [[27], [28], [29], [30], [31]]. Sodium alginate and gelatin were each dissolved in separate containers with 5 ml of phosphate-buffered saline (PBS, pH 7.4) to create two solutions, each with a concentration of 100 mg/ml. Then transglutaminase enzyme was dissolved in 2 ml of distilled water to prepare a 25 mg/ml solution. The transglutaminase solution was then mixed into the gelatin solution to act as a crosslinker for the gelatin. To prepare a 1 M solution of calcium chloride (CaCl2), 73.5 mg of CaCl2 was dissolved in 5 ml of PBS. The alginate solution was combined with the gelatin-transglutaminase mixture. Subsequently, 1 μL of the 1 M CaCl2 solution was added dropwise to the combined solution to serve as a crosslinker for the alginate. All procedures were conducted at room temperature. To ensure effective crosslinking by the transglutaminase enzyme, the final mixture was incubated at 37 °C for 24 h.

#### 2.3.2 Swelling test

Injectable alginate/gelatin was formulated in a plastic vial and weighed after formation. The samples were immersed in 1 ml of PBS (PH = 7.4) and incubated at 37 °C. After 0,1, 2, 5 and 24 h to measure weight changes, PBS was removed from each sample and the excess water inside the vial was removed and the vials were weighed. The inflation percentage was calculated by formula (1):

SwellingRatio%=(Ws−W0)/W0∗100

Where Ws represents the weight of the swollen hydrogel, while W0 represents the initial weight of the hydrogel. All measurements were performed in five replicates [32].

#### 2.3.3 Degradation

An easy, simple and reproducible in vitro method to measure the stability of injectable hydrogels in the laboratory is to monitor their degradation according to the weight loss of the hydrogels initial weight (W0) as a function of incubation time. To check the degradation rate of hydrogel on days 1, 5, 10, 15, 20, 25 and 30, hydrogels were removed from PBS (PH = 7.4) and their weight (Wt) was determined. The mass ratio of the remaining hydrogel was calculated by formula (2) [33]:

RemainingMass(%)=100−(W0–Wt/W0∗100)

### 2.4 Viability assay

To evaluate the metabolic activity of encapsulated EnSCs in hydrogel, the cells were cultured with a density of 1 × 10^5^ inside of injectable alginate/gelatin hydrogel in 96-well plates. After incubation at 37 °C in a humidified atmosphere containing 5% CO2 on days 1, 3 and 5, metabolic activity was assessed by the conventional MTT method. Absorbance values at 570 nm were measured using a SpectraMax® i3 ELISA reader (Molecular Devices, USA) [34].

### 2.5 Animal study

#### 2.5.1 In vivo experimental design

To induce Polycystic Ovary Syndrome (PCOS), a cohort of female rats aged 8 weeks (comprising 40 individuals) received daily injections of letrozole at a dosage of 1 mg/kg for a continuous period of 21 days (30). Concurrently, a separate control group consisting of 5 rats was administered injections of distilled water. During the 21 days of PCOS induction process, the estrous period of rats was checked by crystal violet staining of vaginal smear [35]. All rats were weighed before PCOS induction.

The rats that remained in the diestrum were designated as a model for PCOS, and were further divided into different groups, including Positive control group (sham: n = 5), PCOS group (n = 5), PCOS group receiving hydrogel (PCOS + H, n = 5), PCOS group receiving oral clomiphene citrate (PCOS + C.C, n = 5), PCOS group receiving stem cells (PCOS + S.C, n = 5), PCOS group receiving oral drug clomiphene citrate and stem cells (PCOS + S.C + C.C, n = 5), PCOS group receiving encapsulated stem cells in hydrogel (PCOS + S.C + H, n = 5) and PCOS group receiving encapsulated stem cells in hydrogel and oral drug clomiphene citrate (PCOS + S.C + H + C.C, n = 5). After ensuring the correctness of the induction process, all groups were subjected to surgery. In the groups receiving cells and hydrogel, 1 × 10^6^ cells were encapsulated in 40 μL of hydrogel and injected directly into the left ovary. In cell receiving groups, 1 × 10^6^ cells were suspended in 40 μL of normal saline and injected directly into the left ovary. In the group receiving hydrogel, 40 μL of hydrogel was injected directly into the left ovary. All injections were done with a 30-G needle [36,37]. All intraovarian injections were performed with animals under anesthesia using intraperitoneal injection of a mixture of ketamine (87 mg/kg) and xylazine (13 mg/kg) [38]. All rats were housed with sufficient food and water and at a suitable room temperature. After the surgery, the groups receiving Clomiphene Citrate (2 mg/kg) (30) orally received the drug for one month, and the rest of the groups remained untouched.

#### 2.5.2 Body weight and tissue sampling

Before blood collection, mice were anesthetized by intraperitoneal injection of a mixture of ketamine and xylazine, and their body's weight were recorded. After that, ovarian tissues were completely removed and weighed. Ovaries were used for RNA extraction and H&E staining.

#### 2.5.3 Serum estradiol assay

After one month, the blood of all groups was collected directly from the heart and the serum and buffy Coat were separated. The obtained serum and buffy Coat are stored at −20 °C for measurement of serum levels of hormones and RNA extraction.

#### 2.5.4 Hormonal assay

Blood samples (5 ml) were taken directly from the heart. The blood samples were centrifuged for 8 min (3000r/min) and the supernatant, which is the serum, was collected. The serum concentrations of Anti-Müllerian Hormone (AMH), testosterone, estrogen, Follicle-Stimulating Hormone (FSH), Luteinizing Hormone (LH), and progesterone in the rats were quantified using the enzyme-linked immunosorbent assay (ELISA) technique. The ELISA kits required for these measurements were sourced from Bioassay Technology Laboratory, located in Shanghai, China [39].

#### 2.5.5 Histological analysis

After the rats were sacrificed, both ovaries were separated and fixed in 4% paraformaldehyde. Ovary samples were embedded in paraffin and cut with a thickness of 5 μm. Then tissue sections were stained with H&E [39]. For H&E staining, paraffin sections were first dewaxed with xylene and then treated with an ethanol gradient (anhydrous ethanol, 95% ethanol, 80% ethanol, and 70% ethanol) and distilled water. Deparaffinized tissue sections were stained with hematoxylin staining solution and washed with water. After using the differentiation solution, the samples were washed again with water. The samples were stained with eosin staining solution and washed with water. After soaking the samples in water, they were dehydrated with an ethanol gradient and clarified in xylene. Finally, they were sealed with neutral glue and were observed and photographed with an optical microscope. The histological analysis focused on counting ovarian cysts, corpus luteum, granulosa cells, and immature follicles. Additionally, folliculogenesis was assessed by identifying different follicle types from one in every ten serial sections, with eight sections analyzed per rat. The follicles were classified based on distinct morphological criteria: primordial follicles featured an oocyte surrounded by squamous follicular cells; primary follicles had an oocyte encased by cuboidal granulosa cells (GCs); secondary follicles contained an oocyte with multiple layers of cube-shaped GCs without any cavities; pre-antral follicles had an oocyte with several layers of cube-shaped GCs and small cavities; antral follicles included an oocyte surrounded by cumulus cells (CCs) in a fluid-filled space with multiple GC layers; pre-ovulatory follicles were characterized by an oocyte with CCs and a large cavity around the cumulus-oophorus complex (COC); ovarian cysts were identified by large cavities lined with GCs but without a COC; and the corpus luteum was noted for its multilayered GCs and theca cells (TCs) at the ovary's periphery [40]. This comprehensive approach allowed for a detailed examination of ovarian morphology and the stages of follicle development.

#### 2.5.6 Real-time quantitative PCR

Quantitative real-time PCR was used to measure mRNA expression levels of Transforming growth factor β (TGF-β), Glyceraldehyde 3-phosphate dehydrogenase (Gapdh), Heat shock protein 70 (Hsp70), Interleukin-10 (IL-10), and IL-6. Total RNA extraction was performed using TRIzol reagent (Life Technologies USA) according to the manufacturer's instructions. Two micrograms of total RNA extracted from ovarian tissue was subjected to reverse transcription (RT), and cDNA synthesis was performed using a one-step RT-PCR kit (Takara, Japan). SYBR Green (Toyobo, Japan), RT-PCR amplification and real-time fluorescence detection were performed using an ABI 7300 real-time PCR thermal cycler (ABI, USA) according to the provided protocol. The relative expression of the gene was calculated using the 2^-ΔΔCt^ method and the relative expression levels were normalized to the endogenous expression of the studied genes [41]. The primers used in this study was listed in Table 1. In order to detect the presence of human endometrial stem cells in rat ovarian tissue, the levels of human Gapdh RNA were investigated.

**Table-1:** Primers used for conventional and real time RT-PCR.

Genes	Primer sequence (5″–3″)	Size (pb)	Annealing (◦C)
IL-10 (Rat)	F:AGAGAACCATGGCCCAGAAA R:GGGAGAAATCGATGACAGCG	99 bp	59
IL-6 (Rat)	F:GCAAGAGACTTCCAGCCAGT R: CTGTGAAGTCTCCTCTCCGG	140 bp	59
TGF-B (Rat)	F: CTTCCCCTCCGAAACTGTCT R: ATGGCATCAAGGTACCCACA	105 bp	60
HSP70 (Rat)	F: CTCCGCTTCTCTGCTTCTCT R: CTCGCTGATTGGCCCATG	119 bp	60
Gapdh (Rat)	F: GTTGTGGATCTGACATGCCG R: CCTCAGTGTAGCCCAGGATG	102 bp	59
Gapdh (Human)	F: TGGTATCGTGGAAGGACTCA R: CCAGTAGAGGCAGGGATGAT	132 bp	60

### 2.6 Statistical analysis

Data were expressed as mean ± SEM. All the data were analyzed using one-way ANOVA followed by post hoc Tukey's test using the GraphPad Prism 8.0.2. In all cases, P < 0.05 was considered significant.

## 3 Result and discussion

### 3.1 Endometrial stem cells culturing and characterization

**Fig. 1:**
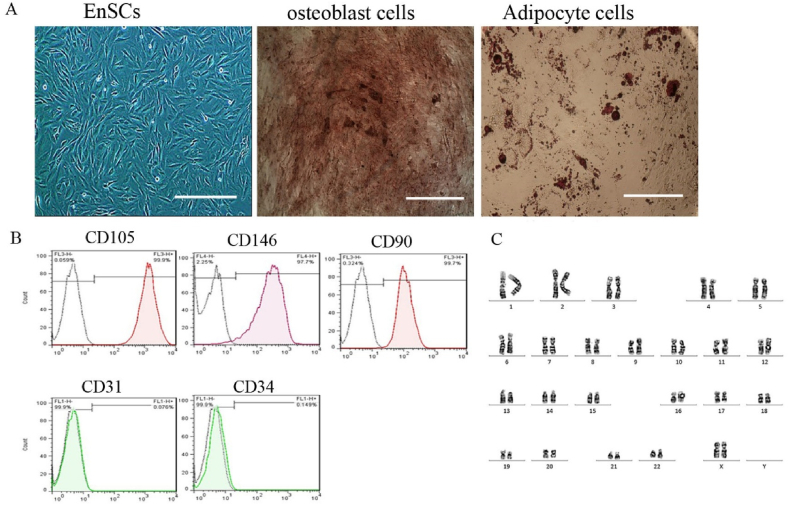
**Endometrial stem cell isolation and characterization. A)** Phase contrast photomicrograph of human endometrial stem cells cultured in the third passage and differentiated cells after 21days of induction into osteoblast and adipocyte cells. The cells usually appeared elongated and spindle-shaped with round nuclei. Scale bare: 100 μm.Microphotographs of isolated EnSCs after 21 days culture in osteogenic and adipogenic induction media stained with alizarin red and oil red o staining. (scale bar is 50 μm). B) Flow cytometric analysis of isolated EnSCs for mesenchymal stem cell markers (CD90, CD105 and CD146), hematopoietic marker (CD34), endothelial marker (CD31). The isolated cells are positive for CD90, CD105 and CD146 and are negative for CD31, CD34. C) Detailed analysis of cultured EnSCs showed normal karyotypes containing 23 pairs of chromosomes.

**Fig. 2:**
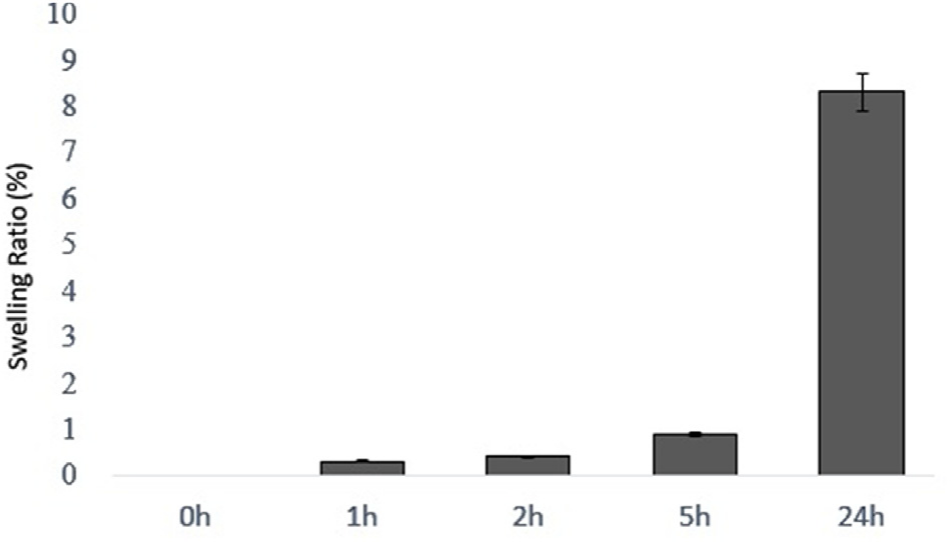
Swelling ratio of alginate/gelatin hydrogel samples in PBS at 37 °C after 1, 3, 5 and 24 h. In this study, 5 samples were examined in terms of swelling test and the average swelling percentage of the samples was reported in different times. (n = 5, mean ± SD).

Endometrial stem cells cultured at the third passage showed uniform spindle shape cells (Fig. 1A). The immunophenotype was assayed based on the flow cytometry analysis for subset of mesenchymal stem cell markers (CD146, CD90 and CD105), hematopoietic marker (CD34) and endothelial marker (CD31). The flow cytometric analysis showed that isolated cells were positive for CD146, CD90, CD105 and were negative for CD31, CD34 (Fig. 1B). When the EnSCs were cultured in osteogenic differentiation media for three weeks, they formed hydroxyapatite crystals, as detected by alizarin red staining, confirming the cells' capacity for osteogenic differentiation. Similarly, the development of neutral lipid vacuoles within the EnSCs, which were cultured in adipogenic media for the same duration, was verified by oil red staining, validating successful adipogenic differentiation (Fig. 1A). The chromosomal integrity of the EnSCs was assessed by G-band karyotyping of cells arrested at metaphase. This analysis confirmed a normal karyotype with 46 chromosomes, including 22 pairs of autosomes and two large X chromosomes (with the inactive X potentially appearing darker in the stain). No chromosomal abnormalities in terms of shape or number were detected (as presented in Fig. 1C) (see Fig. 2).

### 3.2 Hydrogel characterization

#### 3.2.1 Swelling and degradation tests

**Fig. 3:**
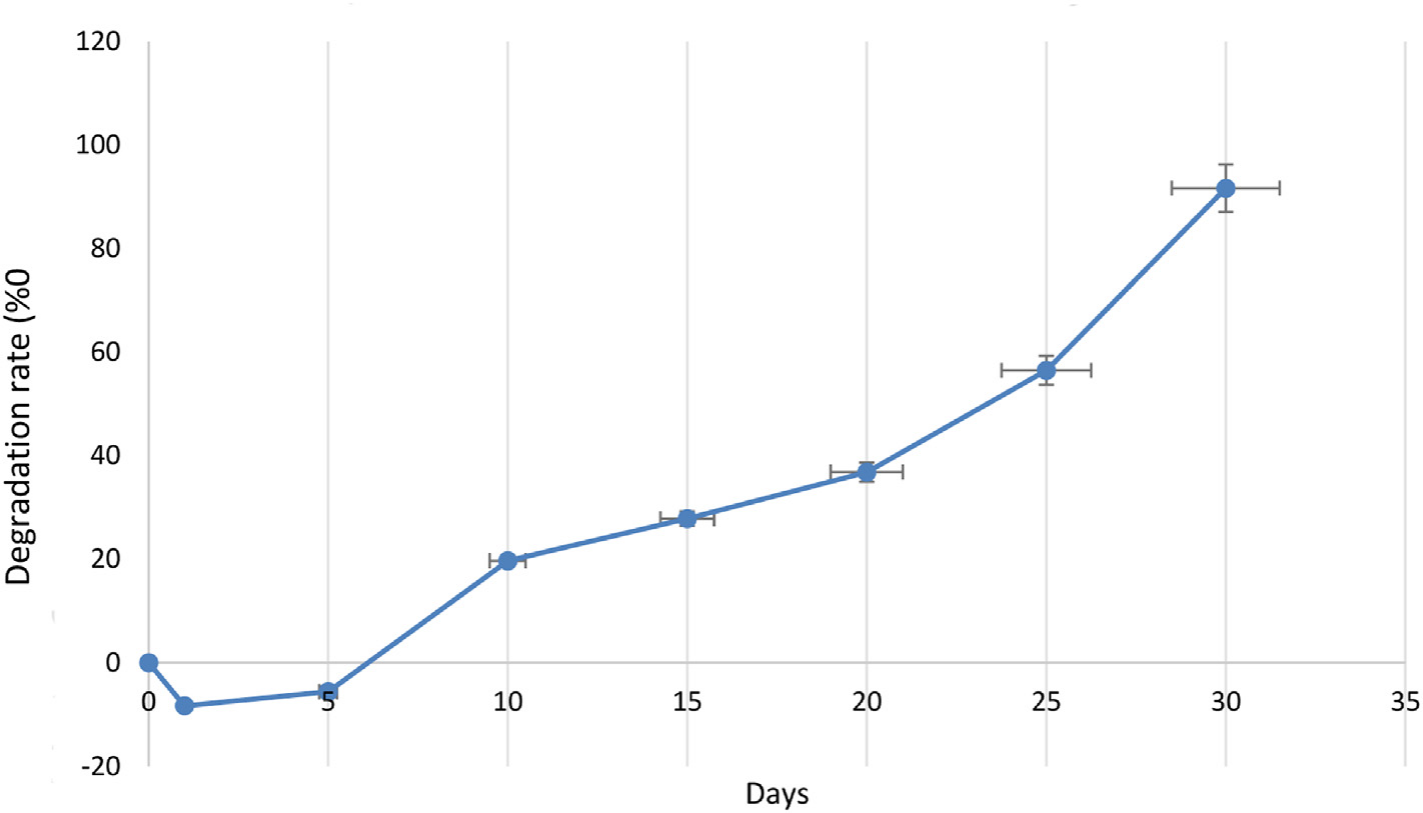
Evaluation of alginate/gelatin hydrogel mass change during continuous immersion in PBS solution on days 1, 5, 10, 15, 20, 25, 30. (n = 3, mean ± SD).

The swelling properties of injectable alginate/gelatin hydrogel were evaluated in PBS solution at 37 °C after 0, 1, 2,5 and 24 h. Five samples of this hydrogel were prepared and their swelling rate was averaged. Hydrogels have a very good ability to absorb water due to their hydrophilicity and high porosity. Alginate/gelatin injectable hydrogel has a swelling rate of 8.32%. The degradation rate of injectable alginate/gelatin hydrogel was investigated during thirty days in PBS (pH = 7.4) at 37 °C. Five samples of this hydrogel were prepared and the average degradation rate was reported. On the first day, due to the absorption of water by the hydrogel, we see an increase in weight, but after that we have an intermittent weight loss, and on the 30th day, the degradability rate reaches 91.64%. The swelling rate of the injectable alginate/gelatin hydrogel is not excessively high, ensuring it does not damage ovarian tissue. Given the degradation rate of the injectable alginate/gelatin hydrogel, it can remain within the ovary for up to one month, degrading slowly (see Fig. 3).

#### 3.2.2 Viability assay

**Fig. 4:**
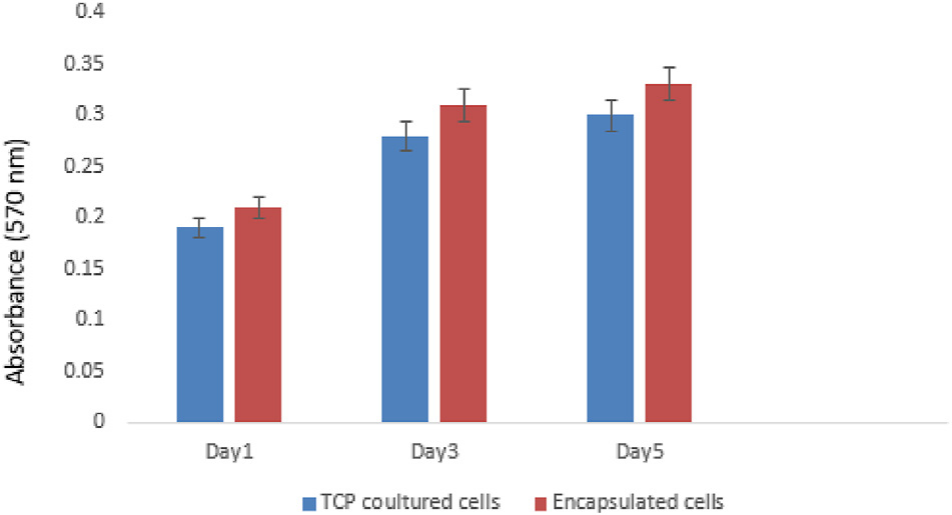
MTT assay analysis the effect of alginate/gelatin hydrogel on the viability of endometrial stem cells was evaluated using the MTT method. MTT assay is based on the ability of mitochondrial dehydrogenase enzymes in living cells to convert MTT to its insoluble form called formazan. Endometrial stem cells were cultured in alginate/gelatin hydrogel, then MTT method was used to evaluate viable cells 1, 3 and 5 days after the start of culture. Despite the good cell viability, there was no statistical difference between groups and days. (n = 3, mean ± SD).

The results of the cytotoxicity and cell viability based on MTT assay for endometrial stem cells in injectable alginate/gelatin hydrogel are shown in Table 1. Based on Fig. 4, the results showed that all hydrogel samples were not toxic, and the viability of encapsulated cells were higher than cells cultured on tissue culture plate (TCP). However, none of the results were significant. Based on our results, the injectable alginate/gelatin hydrogel is biocompatible, and cell viability within it is higher compared to that in two-dimensional culture media. Hydrogels can provide three-dimensional support and attachment sites for stem cells [42], enhancing their adhesion and expansion [43]. Improved adhesion and expansion of stem cells lead to increased production of bioactive factors, which can inhibit inflammation. The increased production of these factors may reduce apoptosis in granulosa cells and decrease the production of inflammatory factors [44].

### 3.3 PCOS rat model confirmation

**Fig. 5:**
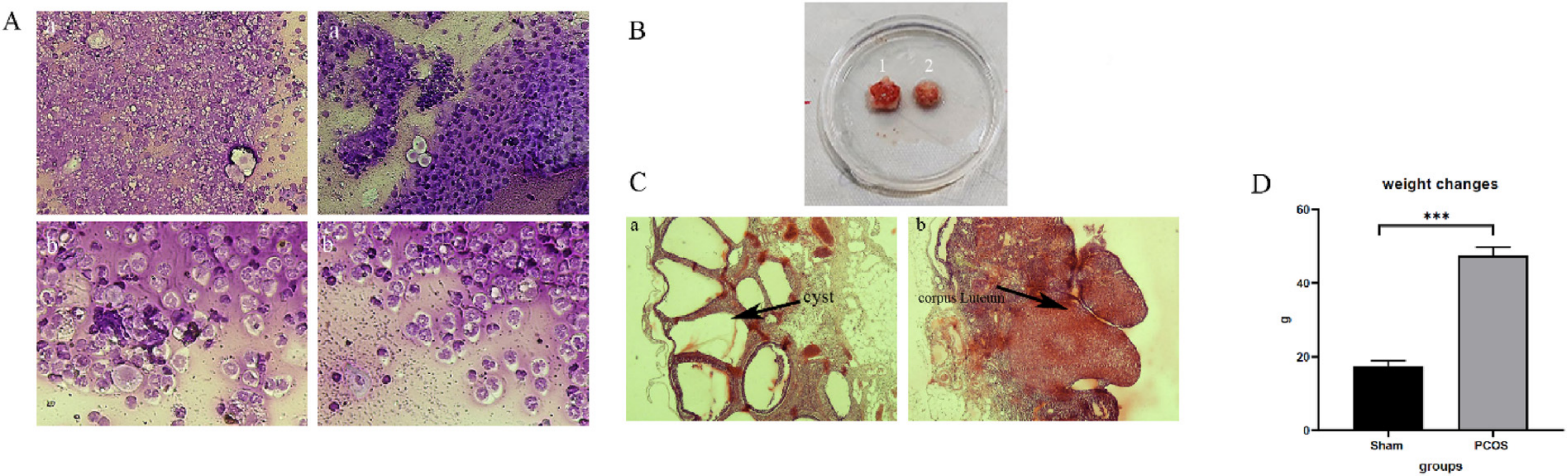
PCOS rat model confirmation. A) Dystrous phase a) Magnification with 20 objective lens b) Magnification with 40 objective lens. B) Ovary volume 1) groups receiving letrozole 2) sham. C) Ovarian cysts a) groups receiving letrozole b) sham. D) weight changes. Changes in the weight of rats in the sham group and the PCOS group were calculated from the start of the PCOS induction process until the end of the process. There was a significant increase in the weight changes of the PCOS group compared to the sham group. (PCOS = polycystic ovary syndrome). Data are presented as the mean ± SD (n = 5, significance level ∗∗∗p < 0.001).

To confirm the PCOS model, after the end of the 21-day treatment period, estrus cyclicity, ovarian histopathology were evaluated. Control rats had estrus cycles of 4–5 days, which included diestrus, proestrus, estrus, and mestruus. PCOS induced rats did not have any dynamic changes in the estrous cycle in the last week of the induction period and were stopped in the diestrus phase. In this phase, mainly leukocytes and a small amount of nucleated epithelial cells can be seen (Fig. 5A). The increase in ovarian volume in the PCOS model compared to the sham group can be seen in Fig. 5B. Additionally, histopathological assessments revealed that the corpus luteum and various stages of follicle development, markers of normal folliculogenesis, were present in the ovaries of the control group (Fig. 5C). In contrast, the ovaries of rats with polycystic ovary syndrome (PCOS) exhibited numerous cystic follicles and lacked evidence of corpus luteum development or ovulation, as shown in Fig. 5C. Rats were weighed at the beginning and end of the PCOS induction period. At the end of the period, rats receiving letrozole (47.4 ± 2.6) gained more weight changes than the control group (17.4 ± 1.6) (Fig. 5D).

The induction of PCOS using letrozole was successful, as evidenced by the female rats exhibiting increased body weight, enlarged ovarian volume, cyst-filled ovaries, and a cessation of the estrous cycle during the diestrus phase. The primary finding of this study is that all five treatment methods—PCOS + C.C, PCOS + S.C, PCOS + S.C + C.C, PCOS + S.C + H, and PCOS + S.C + H + C.C—used to treat PCOS can contribute to the improvement of its symptoms. However, the PCOS + S.C + H and PCOS + S.C + H + C.C groups performed significantly better than the others, suggesting the beneficial role of endometrial stem cells encapsulated within the injectable alginate/gelatin hydrogel in ameliorating PCOS. Moreover, the PCOS + S.C + H + C.C treatment group demonstrated a more effective role in treating PCOS symptoms compared to the PCOS + S.C + H group, indicating that clomiphene citrate can enhance the effect of the encapsulated stem cells within the injectable alginate/gelatin hydrogel. The two treatment groups, PCOS + S.C + H and PCOS + S.C + H + C.C, showed better performance than the PCOS + S.C and PCOS + S.C + C.C groups, highlighting the effective role of the injectable alginate/gelatin hydrogel in improving the function of endometrial stem cells. These two groups also exhibited a more effective role in treating PCOS symptoms than the PCOS + C.C and PCOS + S.C + C.C groups, which suggests that clomiphene citrate alone is not sufficient to fully address PCOS, and that new treatment methods are needed in addition to the common ones.

### 3.4 The effects of encapsulated EnSCs in hydrogel and EnSCs on body weight and ovarian morphometric parameters in PCOS rat model

#### 3.4.1 Body weight changes

**Fig. 6:**
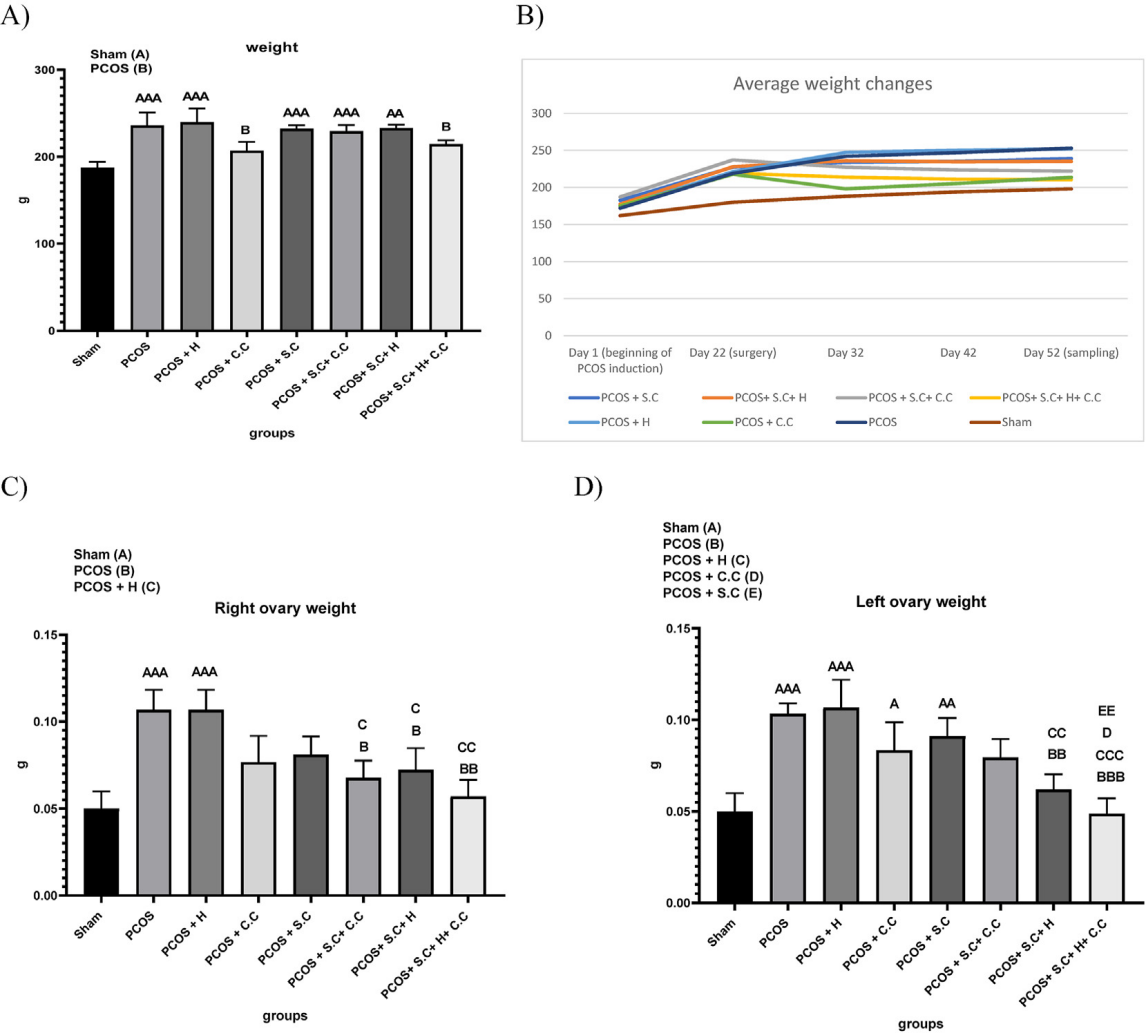
Treatment effects on PCOS animal model. **A)** Weight gain on the last day of treatment compared to the first day of treatment. **B)** The amount of weight changes during the treatment period. **C)** Changes in the weight of the right ovaries. **D)** Change in the weight of the left ovary. (PCOS = polycystic ovary syndrome, S.C = endometrial stem cells, H = alginate/gelatin hydrogel, C.C = clomiphene citrate). Data are presented as the mean ± SD (n = 5, significance level A = ∗p < 0.5, AA = ∗∗p < 0.01, AAA = ∗∗∗p < 0.001, B = ∗p < 0.5, BB = ∗∗p < 0.01, BBB = ∗∗∗p < 0.001, C = ∗p < 0.5, CC = ∗∗p < 0.01, CCC = ∗∗∗p < 0.001, D = ∗p < 0.5, EE = ∗∗p < 0.01).

Rats were weighed continuously throughout the study. The results showed an increase in the weight of rats receiving letrozole. Surgery was performed immediately after the end of the induction period. At the end of the drug treatment period, the rats were weighed. PCOS, PCOS + H, PCOS + S.C, PCOS + S.C + C.C and PCOS + S.C + H groups had significant weight gain compared to the sham group. While the PCOS + C.C and PCOS + S.C + H + C.C groups had significant weight loss compared to the PCOS group (Fig. 6A and . B).

#### 3.4.2 Ovarian weight changes

##### 3.4.2.1 Right ovary

After the drug treatment period, sampling was done immediately and all right ovaries were weighed. The results showed that having PCOS can increase the weight of the right ovary and the PCOS and PCOS + H groups had a significant increase compared to the sham group. PCOS + S.C + C.C, PCOS + S.C + H and PCOS + S.C + H + C.C groups had a significant weight loss in their right ovaries compared to the PCOS and PCOS + H groups, which indicates the effectiveness of treatment methods in reducing the weight of the right ovary in This is the study. However, PCOS + S.C + H + C.C has been more effective than the other two groups (Fig. 6C).

##### 3.4.2.2 Left ovary

After the completion of the drug treatment period, sampling was done immediately and all ovaries on the left side were weighed. The results showed that having polycystic ovary syndrome can increase the weight of the left ovary, and the PCOS and PCOS + H groups had a significant increase compared to the sham group. The treatment methods of PCOS + S.C and PCOS + C.C did not make a significant difference in the weight gain of the left ovary caused by PCOS induction compared to the sham group. However, the treatment method of PCOS + S.C + H + C.C has been more effective than PCOS + S.C + C.C (Fig. 6D).

Obesity is one of the factors involved in the pathogenesis of PCOS [45]. Our results indicate that the combined use of endometrial stem cells encapsulated in an injectable alginate/gelatin hydrogel with clomiphene citrate, as well as the use of clomiphene citrate alone, can lead to weight loss in PCOS patients. However, literature suggests that long-term use of clomiphene citrate may cause weight gain [46]. According to Fig. 11, the use of clomiphene citrate initially causes weight loss, but over time, there is a tendency for weight gain to reoccur. In contrast, the use of endometrial stem cells encapsulated within the injectable alginate/gelatin hydrogel leads to a gradual and sustained reduction in weight. Therefore, if the duration of this study were extended, the use of clomiphene citrate would likely result in significant weight gain, whereas the use of endometrial stem cells within the hydrogel would lead to a significant weight loss compared to the sham group. In patients with PCOS, the ovaries are laden with cysts and become enlarged [47]. Our findings suggest that the use of endometrial stem cells encapsulated within the injectable alginate/gelatin hydrogel can reduce the weight of both the right and left ovaries. This effect is more pronounced in the left ovary, possibly due to the physical presence of the encapsulated endometrial stem cells within the hydrogel.

### 3.5 The effects of encapsulated EnSCs in hydrogel and EnSCs on hormones level in PCOS rat model

#### 3.5.1 LH

**Fig. 7:**
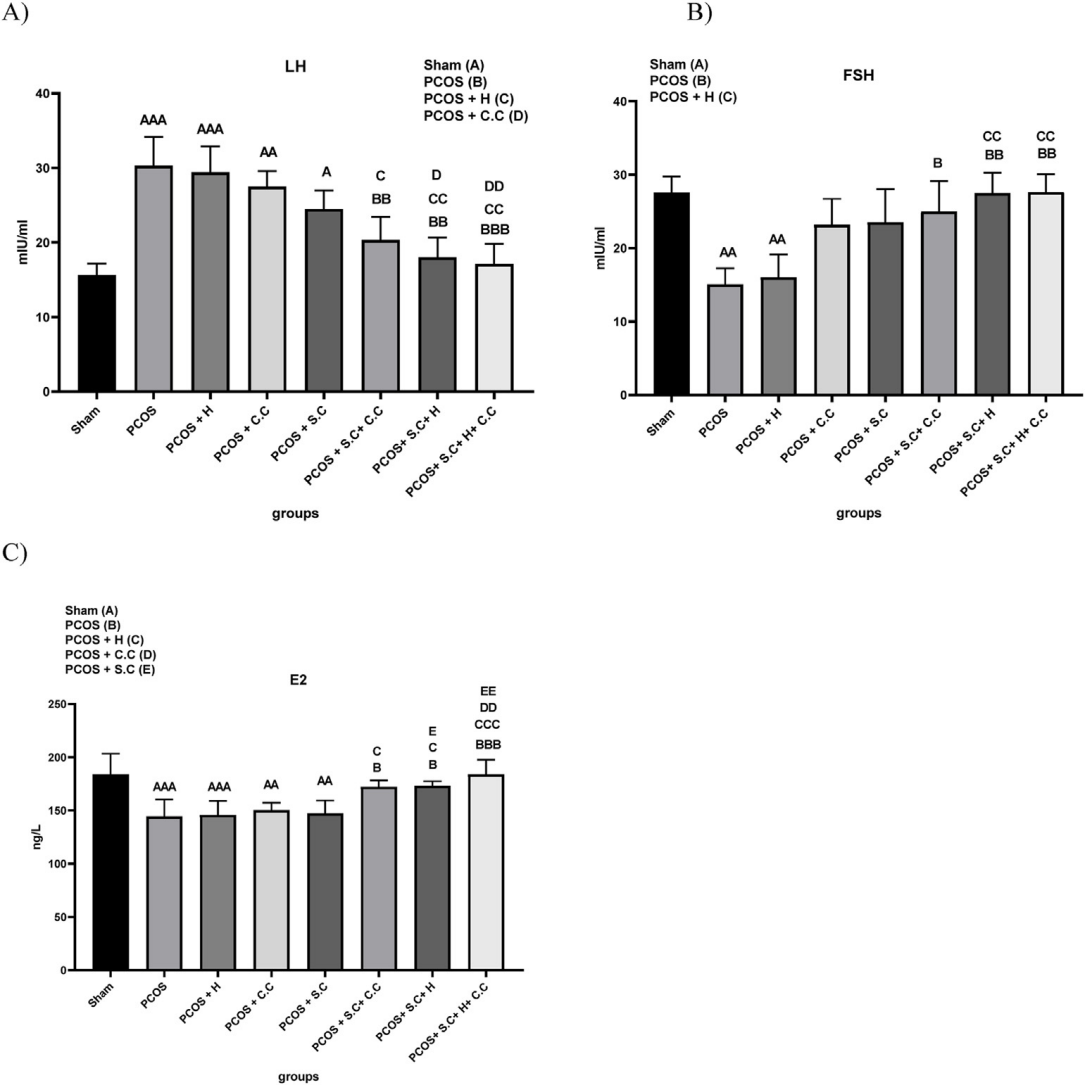
**Treatment effects on hormone level changing in PCOS animal models. A)** Serum level of LH hormone. **B)** Serum level of FSH hormone. **C)** Serum level of estrogen hormone. (PCOS = polycystic ovary syndrome, S.C = endometrial stem cells, H = alginate/gelatin hydrogel, C.C = clomiphene citrate). Data are presented as the mean ± SD (n = 5, significance level A = ∗p < 0.5, AA = ∗∗p < 0.01, AAA = ∗∗∗p < 0.001, B = ∗p < 0.5, BB = ∗∗p < 0.01, BBB = ∗∗∗p < 0.001, C = ∗p < 0.5, CC = ∗∗p < 0.01, CCC = ∗∗∗p < 0.001, D = ∗p < 0.5, DD = ∗∗p < 0.01, E = ∗p < 0.5, EE = ∗∗p < 0.01).

The prepared serum samples were used for LH hormone testing. The results showed that having PCOS can increase the serum level of LH compared to the sham group, and the PCOS and PCOS + H groups had a significant increase compared to the sham group. In the PCOS + S.C + H and PCOS + S.C + H + C.C groups, a significant decrease in LH serum level was observed compared to the PCOS and PCOS + H groups. However, serum LH level did not decrease significantly in PCOS + C.C and PCOS + S.C treatment methods compared to PCOS and PCOS + H groups. Based on the results in the field of LH serum level, the treatment method of PCOS + S.C + H + C.C group is more effective than PCOS + S.C + H treatment group (Fig. 7A).

#### 3.5.2 FSH

FSH hormone test was performed for the prepared serum samples. The results showed that receiving letrozole can reduce the serum level of FSH and the serum level of FSH hormone in the PCOS and PCOS + H groups was significantly reduced compared to the sham group. While PCOS + S.C + C.C, PCOS + S.C + H and PCOS + S.C + H + C.C treatments could significantly improve serum FSH level compared to PCOS group (Fig. 7B).

#### 3.5.3 Esterogen (E2)

In this study, it was shown that rats with PCOS have lower serum estrogen levels than healthy rats. A decrease in estrogen levels indicates hyperandrogenism. In the PCOS and PCOS + H groups, the serum level of estrogen increased significantly compared to the sham group, and PCOS + C.C and PCOS + S.C treatment methods did not significantly affect this increase. While PCOS + S.C + C.C, PCOS + S.C + H and PCOS + S.C + H + C.C treatments improved serum estrogen level compared to PCOS group. However, PCOS + S.C + H + C.C regimen appears to be more effective (Fig. 7B).

#### 3.5.4 Progesterone

**Fig. 8:**
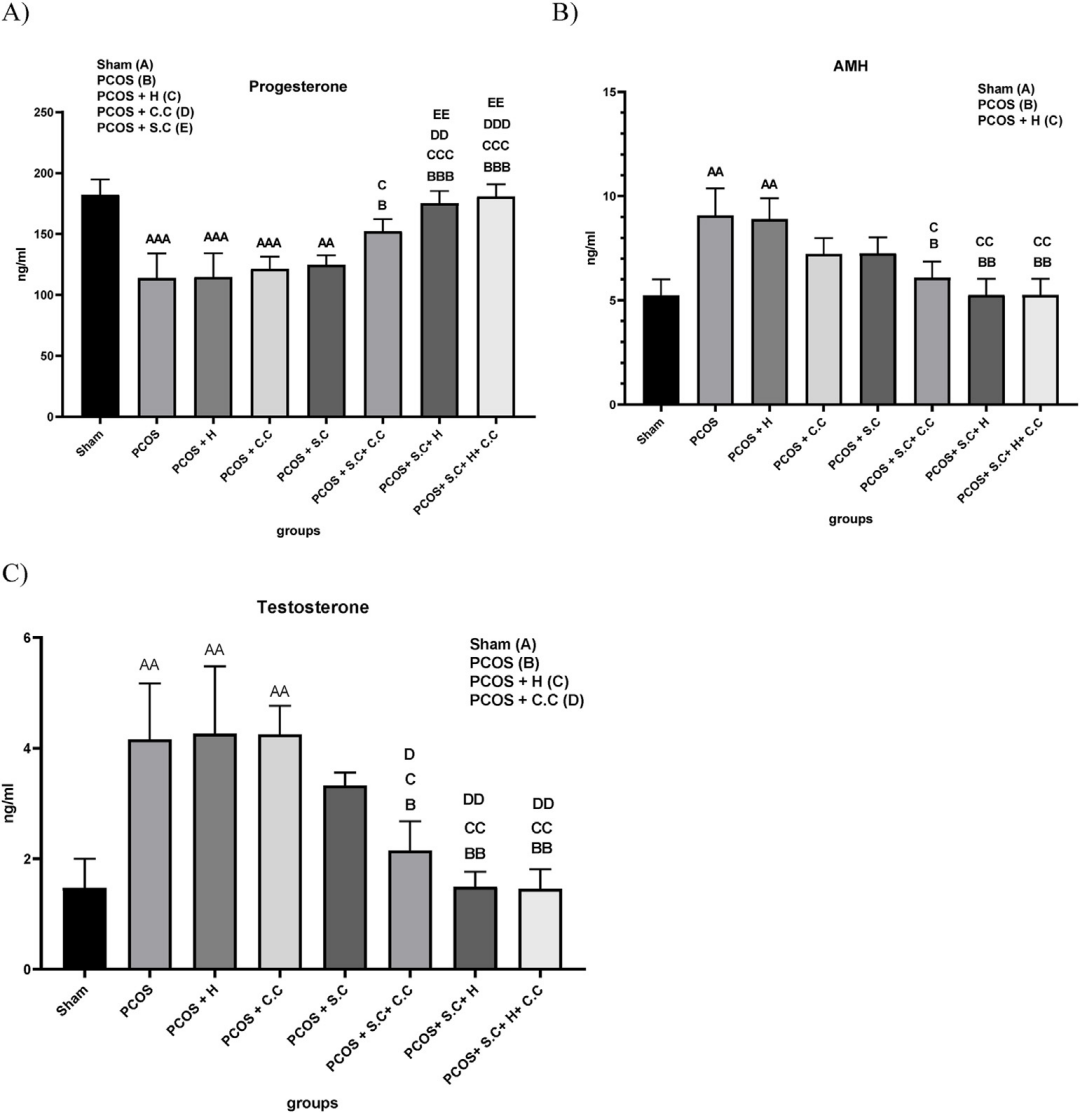
**Treatment effects on hormone level changing in PCOS animal models. A)** Serum level of progesterone hormone. **B)** Serum level of AMH hormone. **C)** Serum level Testosterone hormone. (PCOS = polycystic ovary syndrome, S.C = endometrial stem cells, H = alginate/gelatin hydrogel, C.C = clomiphene citrate). Data are presented as the mean ± SD (n = 5, significance level A = ∗p < 0.5, AA = ∗∗p < 0.01, AAA = ∗∗∗p < 0.001, B = ∗p < 0.5, BB = ∗∗p < 0.01, BBB = ∗∗∗p < 0.001, C = ∗p < 0.5, CC = ∗∗p < 0.01, CCC = ∗∗∗p < 0.001, D = ∗p < 0.5, DD = ∗∗p < 0.01, EE = ∗∗p < 0.01).

In PCOS patients, due to the decrease in corpus luteum formation, the serum level of progesterone also decreases, for this reason, a decrease in progesterone level is observed in the PCOS and PCOS + H groups compared to the sham group. The treatment methods of PCOS + C.C and PCOS + S.C did not have much effect on improving serum progesterone levels. PCOS + S.C + C.C, PCOS + S.C + H and PCOS + S.C + H + C.C treatment methods improved progesterone levels compared to the PCOS group, and in the meantime, the PCOS + S.C + H + C.C treatment method works more efficiently (Fig. 8A).

#### 3.5.5 AMH

One of the ways to diagnose PCOS is to increase the level of AMH. In this study, AMH serum levels increased significantly in PCOS and PCOS + H groups compared to the sham group. The treatment groups PCOS + S.C + C.C, PCOS + S.C + H and PCOS + S.C + H + C.C showed a significant decrease in the serum level of AMH hormone compared to the PCOS group. However, PCOS + C.C, PCOS + S.C treatments did not have much effect on the increase of AMH level (Fig. 8B).

#### 3.5.6 Testosterone

One of the symptoms of PCOS is hyperandrogenism. Testosterone is an indicator androgen that is commonly investigated. In our study, the PCOS and PCOS + H groups showed an increase in serum levels of testosterone compared to the sham group. PCOS + C.C and PCOS + S.C treatment methods did not have much effect on improving serum testosterone levels. In contrast, PCOS + S.C + C.C, PCOS + S.C + H and PCOS + S.C + H + C.C treatment groups showed a significant decrease in serum testosterone levels compared to the PCOS group. However, PCOS + S.C + H and PCOS + S.C + H + C.C groups were more effective in reducing serum testosterone levels (Fig. 8C).

Neuroendocrine abnormalities can initiate or exacerbate the symptoms of polycystic ovary syndrome (PCOS). Neuroendocrine abnormalities can initiate or exacerbate the symptoms of polycystic ovary syndrome (PCOS) [48]. Among these abnormalities are the continuous increase in gonadotropin-releasing hormone (GnRH) pulse frequency and a rise in gonadotropin production, which lead to an abnormal menstrual cycle, a decrease in progesterone levels, and a reduction in estrogen levels. Estrogen and progesterone significantly influence the regulation of GnRH pulses. When levels of progesterone and estrogen decrease, GnRH secretion increases, and PCOS symptoms may worsen. Hyperandrogenemia is associated with increased gonadotropin production [22]. Studies suggest that mesenchymal stem cells can be instrumental in regulating the expression of hormones involved in PCOS. However, the mechanisms by which stem cell transplantation improves the regulation of hormone expression in PCOS and ovarian function require further investigation. Our results indicate that the direct injection of endometrial stem cells increases serum levels of estrogen, anti-Müllerian hormone (AMH), luteinizing hormone (LH), and progesterone, while decreasing serum levels of testosterone and follicle-stimulating hormone (FSH). Nevertheless, the use of endometrial stem cells encapsulated within the injectable alginate/gelatin hydrogel may function more effectively in improving the serum levels of the hormones examined in this study.

### 3.6 Histological analysis

#### 3.6.1 Ovarian cyst formation in right and left ovaries

**Fig. 9:**
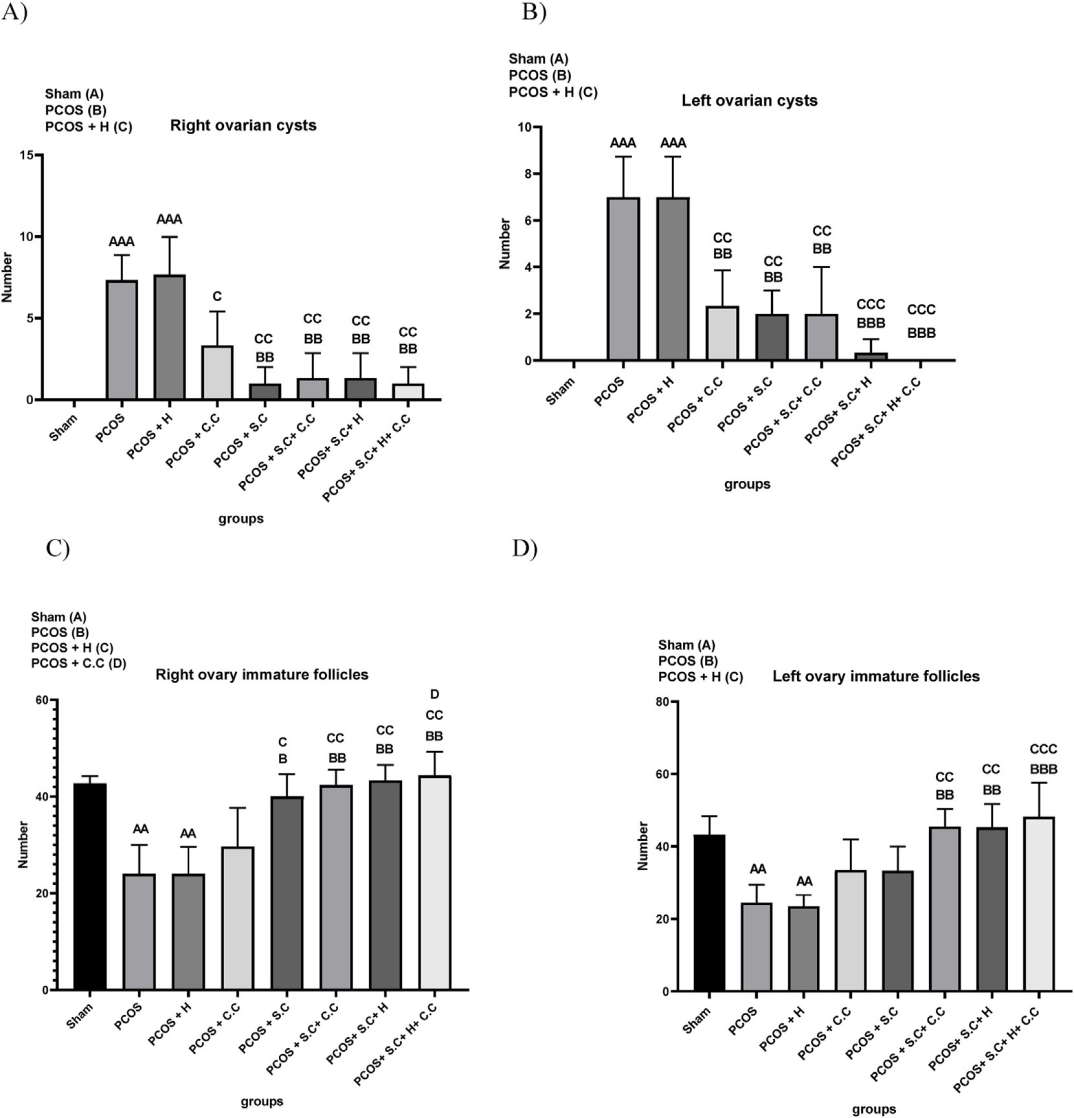
Histological analysis of ovaries after treatment. **A)** Number of right ovarian cysts. **B)** Number of left ovarian cysts. **C)** The number of immature follicles of the right ovary. **D)** The number of immature follicles of the left ovary. (PCOS = polycystic ovary syndrome, S.C = endometrial stem cells, H = alginate/gelatin hydrogel, C.C = clomiphene citrate). Data are presented as the mean ± SD (n = 5, significance level AA = ∗∗p < 0.01, AAA = ∗∗∗p < 0.001, B = ∗p < 0.5, BB = ∗∗p < 0.01, BBB = ∗∗∗p < 0.001, C = ∗p < 0.5, CC = ∗∗p < 0.01, CCC = ∗∗∗p < 0.001, D = ∗p < 0.5).

In women with polycystic ovary syndrome (PCOS), a proliferation of immature follicles often leads to the formation of ovarian cysts. This research observed an abundance of cysts in the right ovaries of the PCOS and PCOS + H groups, while the control group (SH) showed no cyst formation. The treatment groups—PCOS + C.C, PCOS + S.C, PCOS + S.C + C.C, PCOS + S.C + H, and PCOS + S.C + H + C.C—demonstrated a marked reduction in the number of cysts in the right ovary when compared to the untreated PCOS and PCOS + H groups, as shown in Fig. 9A. The left ovary of the PCOS and PCOS + H groups showed a similar pattern with significant presence of cysts, which was absent in the sham group. The aforementioned treatment groups also led to a significant reduction in the number of cysts in the left ovary compared to the PCOS and PCOS + H groups. Notably, two specific treatment combinations—PCOS + S.C + H and PCOS + S.C + H + C.C—were more effective in reducing the number of cysts, as shown in Fig. 9B.

#### 3.6.2 Immature follicles in right and left ovaries

In patients with polycystic ovary syndrome (PCOS), the ratio of luteinizing hormone (LH) to follicle-stimulating hormone (FSH) increases, which interferes with ovulation and the development of mature follicles. This study showed that the number of immature follicles in the right ovary of PCOS and PCOS + H groups was lower compared to the control group (Sham). PCOS + S.C treatment method did not have much effect on the number of immature follicles. However, treatment protocols such as PCOS + S.C, PCOS + S.C + C.C, PCOS + S.C + H, and PCOS + S.C + H + C.C caused a significant increase in immature follicles compared to the PCOS and PCOS + H groups. This indicates an increase in the ovulation process as shown in Fig. 9C.

Similarly, the left ovary of the PCOS and PCOS + H groups decreased the number of immature follicles compared to the sham group. PCOS + C.C and PCOS + S.C treatments did not have much effect on reducing the number of immature follicles. However, the use of PCOS + S.C + C.C, PCOS + S.C + H, and PCOS + S.C + H + C.C treatments resulted in an increase in the number of immature follicles compared to the PCOS and PCOS + H groups, indicating an improvement in the ovulation process, as shown in Fig. 9D.

#### 3.6.3 Corpus luteum in the ovaries

**Fig. 10:**
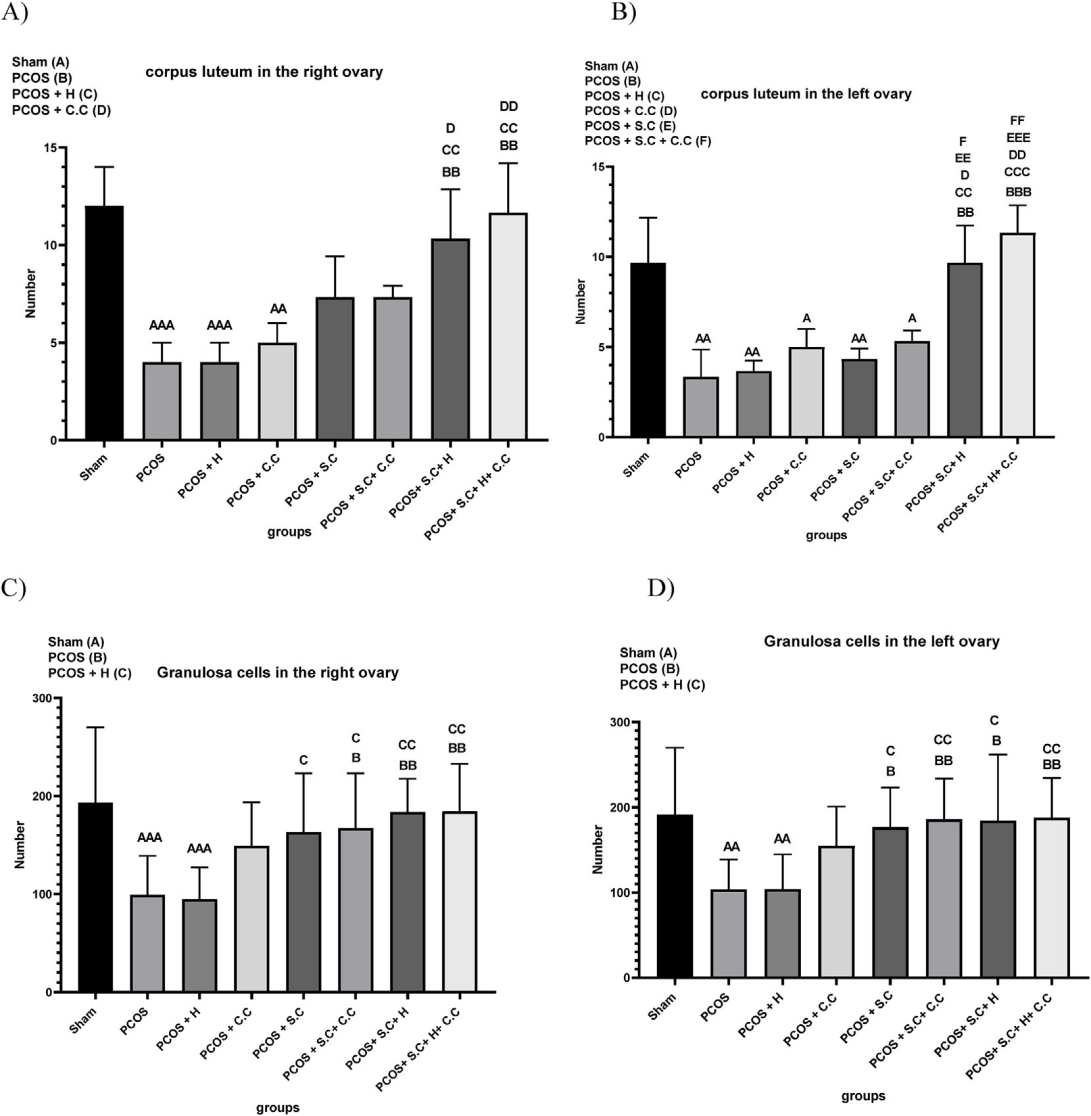
**Histological analysis of ovaries after treatment. A)** The number of corpora lutea of the right ovary. **B)** The number of corpora lutea of the left ovary. **C)** The number of granulosa cells in the right ovary. **D)** The number of granulosa cells in the left ovary. (PCOS = polycystic ovary syndrome, S.C = endometrial stem cells, H = alginate/gelatin hydrogel, C.C = clomiphene citrate). Data are presented as the mean ± SD (n = 5, significance level A = ∗p < 0.5, AA = ∗∗p < 0.01, AAA = ∗∗∗p < 0.001, B = ∗p < 0.5, BB = ∗∗p < 0.01, BBB = ∗∗∗p < 0.001, C = ∗p < 0.5, CC = ∗∗p < 0.01, CCC = ∗∗∗p < 0.001, D = ∗p < 0.5, DD = ∗∗p < 0.01, EE = ∗∗p < 0.1, EEE = ∗∗∗p < 0.01, F = ∗p < 0.5, FF = ∗∗p < 0.01).

Ovulation disorders in women with polycystic ovary syndrome (PCOS) lead to a decrease in the number of corpora lutea in the ovaries. In the current research, a significant decrease in the number of corpora lutea in the right ovary was observed in the PCOS and PCOS + H groups compared to the control group (sham). PCOS + S.C, PCOS + C.C and PCOS + S.C + C.C treatment methods did not have much effect on improving the reduction of corpus luteum caused by PCOS. However, the combined treatment approaches of PCOS + S.C + H and PCOS + S.C + H + C.C significantly increased the number of corpora lutea and enhanced the ovulation process compared to the PCOS and PCOS + H groups, as shown in Fig. 10A. According to Fig. 10A, PCOS + S.C + H + C.C treatment method, it works more efficiently in the field of increasing corpora lutea in the right ovary. Similarly, the number of corpora lutea in the left ovary was decreased in the PCOS and PCOS + H groups compared to the sham group. PCOS + C.C, PCOS + S.C and PCOS + S.C + C.C treatment methods did not have much effect on reducing the number of corpora lutea caused by PCOS induction. However, PCOS + S.C + H and PCOS + S.C + H + C.C treatments, as shown in Fig. 10B, were effective in significantly increasing the number of corpora lutea and facilitating the recovery of the ovulation process. According to Fig. 10B, PCOS + S.C + H + C.C treatment method, it works more efficiently in the field of increasing corpora lutea in the left ovary.

**Fig. 11:**
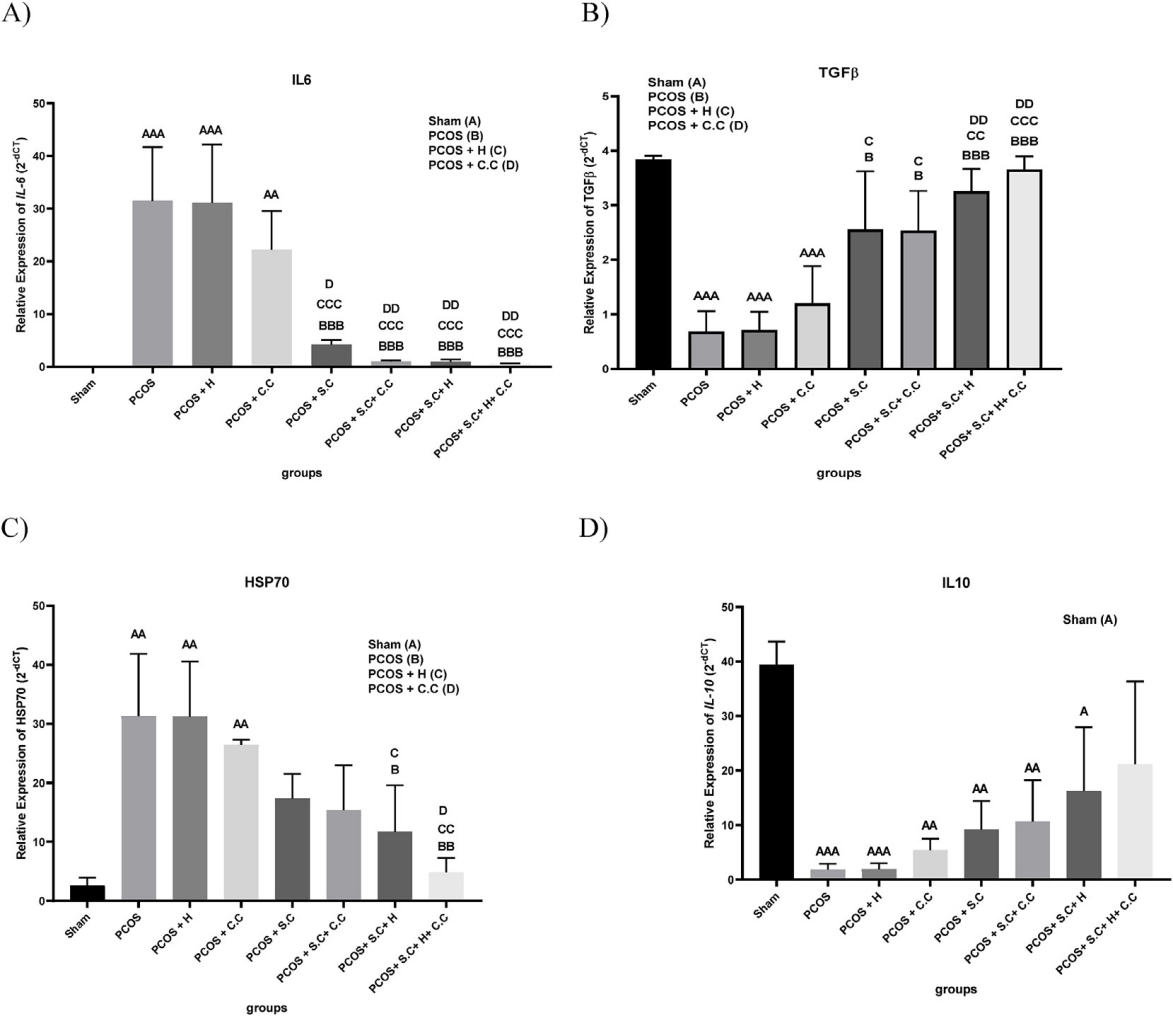
**Real-time quantitative PCR. A)** Serum levels of IL6. **B)** Serum levels of TGFβ. **C)** Serum levels of HSP70. **D)** Serum levels of IL10. (PCOS = polycystic ovary syndrome, S.C = endometrial stem cells, H = alginate/gelatin hydrogel, C.C = clomiphene citrate). Data are presented as the mean ± SD (n = 5, significance level A = ∗p < 0.5, AA = ∗∗p < 0.01, AAA = ∗∗∗p < 0.001, B = ∗p < 0.5, BB = ∗∗p < 0.01, BBB = ∗∗∗p < 0.001, C = ∗p < 0.5, CC = ∗∗p < 0.01, CCC = ∗∗∗p < 0.001, D = ∗p < 0.5, DD = ∗∗p < 0.01).

#### 3.6.4 Granulosa cells in the right and left ovary

Elevated serum insulin levels are a characteristic symptom of polycystic ovary syndrome (PCOS), and research indicates that these increased insulin levels can lead to heightened apoptosis of granulosa cells (GCs) in patients with PCOS. In this particular study, it was observed that the population of granulosa cells in the right ovary of PCOS and PCOS + H groups was reduced compared to the control group (sham). The treatment method of PCOS + C.C did not have a significant effect in improving the decrease in the number of granulosa cells caused by PCOS induction. However, using treatment strategies such as PCOS + S.C + C.C, PCOS + S.C + H and PCOS + S.C + H + C.C, an improvement in the number of granulosa cells in the right ovary was observed compared to the PCOS group as shown in Fig. 10C. Similarly, the left ovary of the PCOS and PCOS + H groups had decreased granulosa cell numbers compared to the sham group. The treatment method of PCOS + C.C did not have much effect in improving the decrease in the number of granulosa cells caused by PCOS induction. PCOS + S.C, PCOS + S.C + C.C, PCOS + S.C + H, and PCOS + S.C + H + C.C treatments were effective in increasing the number of granulosa cells in the left ovary compared to PCOS and PCOS + H groups, as illustrated in Fig. 10D. From a histological perspective, all five treatment methods—PCOS + C.C, PCOS + S.C, PCOS + S.C + C.C, PCOS + S.C + H, and PCOS + S.C + H + C.C—can be effective, potentially leading to an increase in the number of granulosa cells, corpora lutea, and immature follicles, as well as a decrease in the number of cystic follicles in both ovaries. However, endometrial stem cells encapsulated in injectable alginate/gelatin hydrogel appear to offer better therapeutic potential by restoring the function and structure of both ovaries in PCOS rats. Moreover, endometrial stem cells encapsulated within the hydrogel are more effective in restoring the function and structure of the left ovary due to the physical presence of the cells.

The impact of PCOS on the number of immature follicles remains unclear in various studies. Some research suggests that the number of immature follicles increases due to ovulatory disorders [5], while other studies propose that the conversion of a large number of immature follicles into cysts and the constant ovarian reserve should result in a reduced number of immature follicles [49]. The beneficial effects of using stem cells in models of ovarian dysfunction on the inflammation of damaged ovarian tissues have been documented [50].

### 3.7 Real-time quantitative PCR

One of the important factors involved in the pathogenesis of PCOS is the change in the expression of inflammatory factors. In the PCOS group, the expression level of IL6 and HSP70 increased compared to the sham group, while the expression level of IL10 and TGF-β decreased in the PCOS group compared to the sham group. The results indicate that in the PCOS + S.C, PCOS + S.C + C.C, PCOS + S.C + H and PCOS + S.C + H + C.C groups, the levels of TGF-β and IL6 improved due to cell therapy (Fig. 11A and B). Also, in PCOS + S.C + H and PCOS + S.C + H + C.C groups, cells encapsulated in hydrogel were able to improve HSP70 levels (Fig. 11C). Treatment methods had no significant effect on IL10 levels (Fig. 11D). In addition, the presence of endometrial stem cells inside the left ovary of PCOS + S.C + H and PCOS + S.C + H + C.C groups was confirmed.

Stem cells have been shown to restore ovarian function by reducing pro-inflammatory cytokines [51]. Studies have shown that changing the expression of transforming growth factor beta 1 (TGF-β1) in the ovaries increases the apoptosis of granulosa cells in the PCOS model. Our study suggests that the transplantation of endometrial stem cells (EnSCs) may facilitate the improvement of TGF-β1 and other inflammatory factors [22,52]. We demonstrated that transplanting EnSCs, with or without the injectable alginate/gelatin hydrogel, improves gonadotropin levels and increases the presence of healthy follicles in a PCOS rat model. Articles show that in people with PCOS, serum HSP70 level has a negative correlation with Th17, IL-10 and TGF-β levels. In contrast, HSP70 levels were significantly positively correlated with IL-6 and Treg levels [53]. Therefore, EnSCs can play a role in the treatment of PCOS by improving the levels of inflammatory factors. This effect can be intensified by encapsulating cells in an injectable alginate/gelatin hydrogel.

## 4 Conclusion

In this study, we leveraged numerous capabilities, including the anti-inflammatory properties and hormonal expression regulation of stem cells, as well as the porosity, three-dimensional structure, injectability, and homeostasis of the scaffold. Although this study yielded positive outcomes in the transplantation of endometrial stem cells (EnSCs) with or without injectable alginate/gelatin hydrogel, the underlying mechanisms and potential risks remain elusive and warrant further investigation. The current study presents the outcomes of various therapeutic approaches that may prove beneficial for treating polycystic ovary syndrome (PCOS) and advancing regenerative medicine. This research marks the inaugural use of EnSCs transplantation with or without injectable alginate/gelatin hydrogel for PCOS treatment. The findings indicate that EnSCs transplantation, both with and without the hydrogel, may contribute to the amelioration of PCOS symptoms.

## Ethics approval and consent to participate

All animal experiments were approved by the Ethics Committee of Tehran University of Medical Sciences (Ethical Approval No. IR.TUMS.AEC.1401.104) in accordance with institutional and international guidelines.

## Consent for publication

Not Applicable.

## Availability of data and materials

The datasets used and analyzed during the current study available from the corresponding author on reasonable request.

## Funding

The authors thank 10.13039/501100004484Tehran University of Medical Sciences (Grant No.1401-3-148-62403) for supporting this research.

## Credit authorship contribution statement

Fatemeh Kouchakzadeh: Methodology, Investigation, Writing-Original draft Somayeh Ebrahimi-Barough: Supervisions, Conceptualization, Reviewing and Editing Behrouz Aflatoonian and Jalal Golzadeh: Cell culture and analysis.

Jafar Ai: Reviewing and editing Fahime Mazaheri, Fateme Montazeri, Fatemeh Hajizadeh-Tafti: Data analysis and Resources and Software Reza Naser and Masoumeh Sepehri: Scaffold designing and analyzing.

Seyed Mehdi Kalantar: Analysis of animal study results.

## Declaration of competing interest

The authors declare that they have no competing interests.
